# Scheelite-type NaEr(MoO_4_)_2_

**DOI:** 10.1107/S160053681001264X

**Published:** 2010-04-10

**Authors:** Dan Zhao, Feifei Li, Wendan Cheng, Hao Zhang

**Affiliations:** a Department of Physics and Chemistry, Henan Polytechnic University, Jiaozuo, Henan 454000, People’s Republic of China; b State Key Laboratory of Structural Chemistry, Fujian Institute of Research on the Structure of Matter, Chinese Academy of Sciences, Fuzhou, Fujian 350002, People’s Republic of China

## Abstract

Explorations of the *A* ^1+^–*RE* ^3+^–Mo^6+^–O^2−^ (*A* ^1+^ is an alkali metal cation, *RE* ^3+^ is a rare-earth metal cation) quaternary systems prepared by the high-temperature solution growth method led to the title structure, sodium erbium bis­(molyb­date), NaEr(MoO_4_)_2_. It is isostructural to the scheelite structure (CaWO_4_) and is composed of [MoO_4_]^2−^ tetra­hedra with 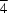 symmetry and [(Na/Er)O_8_]^14−^ polyhedra. The [(Na/Er)O_8_]^14−^ polyhedron is a distorted tetra­gonal anti­prism, also with 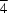 symmetry, with statistically mixed Na/Er atoms at its centre. There are two sets of Na/Er—O bond lengths [2.420 (4) and 2.435 (3) Å], but just one set of Mo—O bond lengths [1.774 (4) Å].

## Related literature

For the structures, properties and applications of the alkali rare-earth tungstates and molybdates with the general formula *A* ^1+^ *RE* ^3+^(*M* ^6+^O_4_)_2_ (*A* ^1+^ is an alkali metal cation, *RE* ^3+^ is a rare-earth metal cation, *M* ^6+^ is Mo^6+^ or W^6+^), see: Huang *et al.* (2006 ▶); Klevtsova (1975 ▶); Klevtsova *et al.* (1972 ▶); Kolitsch (2001 ▶); Kuzmicheva *et al.* (2005 ▶); Li *et al.* (2006 ▶); Morozov *et al.* (2006 ▶); Stevens *et al.* (1991 ▶); Zhao *et al.* (2010 ▶). For the scheelite (CaWO_4_) structure, see: Sillen & Nylander (1943 ▶).

## Experimental

### 

#### Crystal data

NaEr(MoO_4_)_2_
*M* *_r_* = 510.13
Tetragonal, 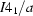

*a* = 5.1816 (8) Å
*c* = 11.288 (3) Å
*V* = 303.07 (11) Å^3^
*Z* = 2
Mo *K*α radiation
μ = 17.87 mm^−1^
*T* = 173 K
0.08 × 0.04 × 0.04 mm

#### Data collection

Rigaku Saturn70 CCD diffractometer
Absorption correction: multi-scan (rescaled *SADABS*; Sheldrick, 1997 ▶) *T*_min_ = 0.263, *T*_max_ = 0.489
520 measured reflections
172 independent reflections
106 reflections with *I* > 2σ(*I*)
*R*_int_ = 0.026

#### Refinement

*R*[*F* ^2^ > 2σ(*F* ^2^)] = 0.032
*wR*(*F* ^2^) = 0.089
*S* = 0.84
172 reflections
15 parameters
Δρ_max_ = 1.12 e Å^−3^
Δρ_min_ = −1.15 e Å^−3^

### 

Data collection: *CrystalClear* (Rigaku, 2004 ▶); cell refinement: *CrystalClear*; data reduction: *CrystalClear*; program(s) used to solve structure: *SHELXS97* (Sheldrick, 2008 ▶) and *PLATON* (Spek, 2009 ▶); program(s) used to refine structure: *SHELXL97* (Sheldrick, 2008 ▶); molecular graphics: *DIAMOND* (Brandenburg, 2004 ▶); software used to prepare material for publication: *SHELXTL* (Sheldrick, 2008 ▶).

## Supplementary Material

Crystal structure: contains datablocks I, global. DOI: 10.1107/S160053681001264X/fb2187sup1.cif

Structure factors: contains datablocks I. DOI: 10.1107/S160053681001264X/fb2187Isup2.hkl

Additional supplementary materials:  crystallographic information; 3D view; checkCIF report

## Figures and Tables

**Fig. 1.**
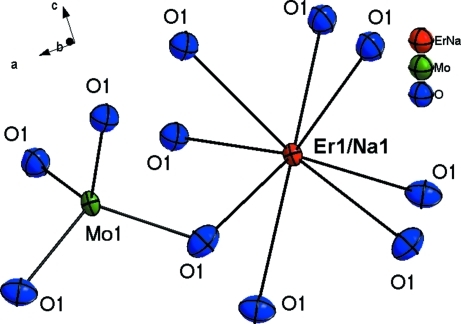
Section of the structure of NaEr(MoO4)2 with the atom labelling scheme. The displacement ellipsoids are drawn at the 50% probability level.

**Fig. 2.**
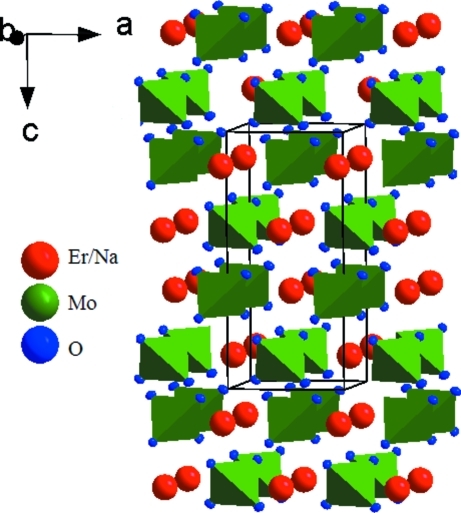
View of the crystal structure of NaEr(MoO4)2. The [MoO4]2- tetrahedra are shown in green.

## supplementary crystallographic information

### Comment

Alkali rare-earth bis(molybdates) with the general formula *A*^1+^*RE*^3+^(*M*O_4_)_2_ (*A*^I^ is an alkali-metal cation, *RE*^3+^ is a rare-earth metal cation, *M* is Mo^6+^ or W^6+^) have been the subject of interest for many decades, mainly due to their applications as suitable host materials for fluorescence (Kuzmicheva *et al.*, 2005; Morozov *et al.*, 2006; Li *et al.*, 2006). Some of these crystals are isostructural to scheelite (CaWO_4_, *I*4_1_/*a*; Sillen & Nylander, 1943), such as NaLa(MoO_4_)_2_ (Stevens *et al.*, 1991), LiNd(MoO_4_)_2_ (Kolitsch, 2001), LiNd(WO_4_)_2_ (Huang *et al.*, 2006) and LiDy(WO_4_)_2_ (Zhao *et al.*, 2010).

In difference to CaWO_4_ with one cation species only, the cations *A*^1+^ and *RE*^3+^ are statistically disordered. Within alkali rare-earth bis(molybdates), different structures from the scheelite type have also been reported, such as LiLa(MoO_4_)_2_ (*Pbca*; Klevtsova, 1975) and CsDy(MoO_4_)_2_ (*Pccm*; Klevtsova *et al.* 1972).

The X-ray diffraction analysis has shown that the title compound NaEr(MoO_4_)_2_ is isostructural with the scheelite. In the title structure, Na and Er atoms are disordered over the same 4*a* site while Mo atoms reside on 4*b* site. The structure of NaEr(MoO_4_)_2_ may be regarded as composed of [MoO_4_]^2-^ tetrahedra and of [(Na/Er)O_8_]^14-^ polyhedra (each in the form of a distorted tetragonal antiprism) that share the oxygens (Fig. 2). Each oxygen of the [MoO_4_]^2-^ tetrahedron is shared by the different Na/Er polyhedron and each oxygen of the [(Na/Er)O_8_]^14-^ polyhedron is shared by the different [MoO_4_]^2-^ tetrahedron.

### Experimental

Single crystals of NaEr(MoO_4_)_2_ have been prepared by the high temperature solution growth (HTSG) method in air. A powder mixture of Na_2_CO_3_ (0.4418 g), Er_2_O_3_ (0.2657 g) and MoO_3_ (2.000 g) at the molar ratio of Na:Er:Mo = 6:1:10 was first ground in an agate mortar and then transferred to a platinum crucible. The sample was gradually heated in air at 1173 K for 24 h. In this stage, the reagents were completely melted. After that, the intermediate product was slowly cooled to 673 K at the rate of 2 Kh^-1^. It was kept at 673 for another 10 h and then quenched to room temperature. The obtained crystals were light-red and of the prismatical shape. The dimensions of the used sample were typical for the grown crystals in this batch.

### Refinement

The Na and Er atoms are in substitutional disorder in the crystal structure. The tentative refinement that included the corresponding occupancy factors for the disordered Na/Er yielded Na1 : Er1 = 0.501 (2) : 0.499 (2). (The atomic positional and anisotropic displacement parameters of Na1 and Er1 atoms were constrained to be identical by using EADP and EXYZ constraint instructions (SHELXL-97; Sheldrick, 2008).) Therefore the ratio of Na and Er was fixed to 1:1 in the final model with the constrained positional and the displacement parameters of na and Er as given above. The highest peak in the difference electron density map equals to 1.12 e/Å^3^ at the distance of 0.83 Å from Na1/Er1 site while the deepest hole equals to -1.15 e/Å^3^ at the distance of 1.39 Å from Na1/Er1 site, too.

### Crystal data

**Table:**

NaEr(MoO_4_)_2_	*D*_x_ = 5.590 Mg m^−^^3^
*M**_r_* = 510.13	Mo *K*α radiation, λ = 0.71073 Å
Tetragonal, *I*4_1_/*a*	Cell parameters from 365 reflections
Hall symbol: -I 4ad	θ = 4.3–27.3°
*a* = 5.1816 (8) Å	µ = 17.87 mm^−^^1^
*c* = 11.288 (3) Å	*T* = 173 K
*V* = 303.07 (11) Å^3^	Prism, red
*Z* = 2	0.08 × 0.04 × 0.04 mm
*F*(000) = 454	

### Data collection

**Table:**

Rigaku Saturn70 CCD diffractometer	172 independent reflections
Radiation source: fine-focus sealed tube	106 reflections with *I* > 2σ(*I*)
confocal	*R*_int_ = 0.026
Detector resolution: 28.5714 pixels mm^-1^	θ_max_ = 27.5°, θ_min_ = 4.3°
ω scans	*h* = −2→6
Absorption correction: multi-scan (rescaled *SADABS*; Sheldrick, 1997)	*k* = −5→6
*T*_min_ = 0.263, *T*_max_ = 0.489	*l* = −14→14
520 measured reflections	

### Refinement

**Table:**

Refinement on *F*^2^	Primary atom site location: structure-invariant direct methods
Least-squares matrix: full	Secondary atom site location: difference Fourier map
*R*[*F*^2^ > 2σ(*F*^2^)] = 0.032	*w* = 1/[σ^2^(*F*_o_^2^) + (0.0639*P*)^2^] where *P* = (*F*_o_^2^ + 2*F*_c_^2^)/3
*wR*(*F*^2^) = 0.089	(Δ/σ)_max_ < 0.001
*S* = 0.84	Δρ_max_ = 1.12 e Å^−^^3^
172 reflections	Δρ_min_ = −1.14 e Å^−^^3^
15 parameters	Extinction correction: *SHELXL97* (Sheldrick, 2008), Fc^*^=kFc[1+0.001xFc^2^λ^3^/sin(2θ)]^-1/4^
0 restraints	Extinction coefficient: 0.055 (5)
9 constraints	

### Special details

**Table:**

Geometry. All esds (except the esd in the dihedral angle between two l.s. planes) are estimated using the full covariance matrix. The cell esds are taken into account individually in the estimation of esds in distances, angles and torsion angles; correlations between esds in cell parameters are only used when they are defined by crystal symmetry. An approximate (isotropic) treatment of cell esds is used for estimating esds involving l.s. planes.
Refinement. Refinement of *F*^2^ against ALL reflections. The weighted *R*-factor wR and goodness of fit *S* are based on *F*^2^, conventional *R*-factors *R* are based on *F*, with *F* set to zero for negative *F*^2^. The threshold expression of *F*^2^ > σ(*F*^2^) is used only for calculating *R*-factors(gt) etc. and is not relevant to the choice of reflections for refinement. *R*-factors based on *F*^2^ are statistically about twice as large as those based on *F*, and *R*- factors based on ALL data will be even larger.

### Fractional atomic coordinates and isotropic or equivalent isotropic displacement parameters (Å^2^)

**Table:**

	*x*	*y*	*z*	*U*_iso_*/*U*_eq_	Occ. (<1)
Er1	0.0000	0.2500	0.1250	0.0081 (5)	0.50
Na1	0.0000	0.2500	0.1250	0.0081 (5)	0.50
Mo1	0.5000	0.7500	0.1250	0.0086 (5)	
O1	0.2568 (6)	0.5968 (6)	0.0397 (3)	0.0204 (12)	

### Atomic displacement parameters (Å^2^)

**Table:**

	*U*^11^	*U*^22^	*U*^33^	*U*^12^	*U*^13^	*U*^23^
Er1	0.0070 (6)	0.0070 (6)	0.0102 (8)	0.000	0.000	0.000
Na1	0.0070 (6)	0.0070 (6)	0.0102 (8)	0.000	0.000	0.000
Mo1	0.0067 (6)	0.0067 (6)	0.0124 (8)	0.000	0.000	0.000
O1	0.025 (2)	0.017 (2)	0.019 (2)	0.0012 (15)	−0.0045 (14)	−0.0007 (17)

### Geometric parameters (Å, °)

**Table:**

Er1—O1^i^	2.420 (4)	Er1—O1^vii^	2.435 (3)
Er1—O1^ii^	2.420 (4)	Mo1—O1^viii^	1.774 (4)
Er1—O1^iii^	2.420 (4)	Mo1—O1^ix^	1.774 (4)
Er1—O1^iv^	2.420 (4)	Mo1—O1	1.774 (4)
Er1—O1^v^	2.435 (3)	Mo1—O1^x^	1.774 (4)
Er1—O1^vi^	2.435 (3)	O1—Na1^iii^	2.420 (4)
Er1—O1	2.435 (3)	O1—Er1^iii^	2.420 (4)
			
O1^i^—Er1—O1^ii^	79.63 (16)	O1^v^—Er1—O1^vii^	99.01 (7)
O1^i^—Er1—O1^iii^	126.16 (10)	O1^vi^—Er1—O1^vii^	99.01 (7)
O1^ii^—Er1—O1^iii^	126.16 (10)	O1—Er1—O1^vii^	133.38 (18)
O1^i^—Er1—O1^iv^	126.16 (10)	O1^i^—Er1—Er1^xi^	38.03 (7)
O1^ii^—Er1—O1^iv^	126.16 (10)	O1^ii^—Er1—Er1^xi^	69.88 (9)
O1^iii^—Er1—O1^iv^	79.62 (16)	O1^iii^—Er1—Er1^xi^	159.67 (8)
O1^i^—Er1—O1^v^	75.78 (12)	O1^iv^—Er1—Er1^xi^	101.19 (8)
O1^ii^—Er1—O1^v^	68.76 (7)	O1^v^—Er1—Er1^xi^	37.75 (8)
O1^iii^—Er1—O1^v^	152.76 (16)	O1^vi^—Er1—Er1^xi^	101.99 (9)
O1^iv^—Er1—O1^v^	73.67 (7)	O1—Er1—Er1^xi^	85.52 (9)
O1^i^—Er1—O1^vi^	68.76 (7)	O1^vii^—Er1—Er1^xi^	131.38 (8)
O1^ii^—Er1—O1^vi^	75.78 (12)	O1^i^—Er1—Na1^xi^	38.03 (7)
O1^iii^—Er1—O1^vi^	73.67 (7)	O1^ii^—Er1—Na1^xi^	69.88 (9)
O1^iv^—Er1—O1^vi^	152.76 (16)	O1^iii^—Er1—Na1^xi^	159.67 (8)
O1^v^—Er1—O1^vi^	133.38 (18)	O1^iv^—Er1—Na1^xi^	101.19 (8)
O1^i^—Er1—O1	73.67 (7)	O1^v^—Er1—Na1^xi^	37.75 (8)
O1^ii^—Er1—O1	152.76 (16)	O1^vi^—Er1—Na1^xi^	101.99 (9)
O1^iii^—Er1—O1	75.78 (12)	O1—Er1—Na1^xi^	85.52 (9)
O1^iv^—Er1—O1	68.76 (7)	O1^vii^—Er1—Na1^xi^	131.38 (8)
O1^v^—Er1—O1	99.01 (7)	O1^viii^—Mo1—O1^ix^	114.2 (2)
O1^vi^—Er1—O1	99.01 (7)	O1^viii^—Mo1—O1	107.15 (11)
O1^i^—Er1—O1^vii^	152.76 (16)	O1^ix^—Mo1—O1	107.15 (11)
O1^ii^—Er1—O1^vii^	73.67 (7)	O1^viii^—Mo1—O1^x^	107.15 (11)
O1^iii^—Er1—O1^vii^	68.76 (7)	O1^ix^—Mo1—O1^x^	107.15 (11)
O1^iv^—Er1—O1^vii^	75.78 (12)	O1—Mo1—O1^x^	114.2 (2)

Symmetry codes: (i) −*y*+3/4, *x*+1/4, *z*+1/4; (ii) *y*−3/4, −*x*+1/4, *z*+1/4; (iii) −*x*, −*y*+1, −*z*; (iv) *x*, *y*−1/2, −*z*; (v) *y*−1/4, −*x*+1/4, −*z*+1/4; (vi) −*y*+1/4, *x*+1/4, −*z*+1/4; (vii) −*x*, −*y*+1/2, *z*; (viii) *y*−1/4, −*x*+5/4, −*z*+1/4; (ix) −*y*+5/4, *x*+1/4, −*z*+1/4; (x) −*x*+1, −*y*+3/2, *z*; (xi) −*x*+1/2, −*y*+1/2, −*z*+1/2.
